# Case report of a cardiac Harlequin syndrome—electrical storm during venoarterial extracorporeal membrane oxygenation

**DOI:** 10.1093/ehjcr/ytaf059

**Published:** 2025-02-05

**Authors:** Maria Heinrich, Lars S Maier, Thomas Müller, Matthias Lubnow, Alexander Dietl

**Affiliations:** Department of Internal Medicine II, University Hospital Regensburg, Regensburg D-93053, Germany; Department of Internal Medicine II, University Hospital Regensburg, Regensburg D-93053, Germany; Department of Internal Medicine II, University Hospital Regensburg, Regensburg D-93053, Germany; Department of Internal Medicine II, University Hospital Regensburg, Regensburg D-93053, Germany; Department of Internal Medicine II, University Hospital Regensburg, Regensburg D-93053, Germany

**Keywords:** Differential hypoxia, Harlequin syndrome, V-AV-ECMO, Case report

## Abstract

**Background:**

In sepsis-induced cardiogenic shock, venoarterial extracorporeal membrane oxygenation (VA-ECMO) can improve survival. Simultaneous acute respiratory distress syndrome (ARDS) increases the risk of differential hypoxia (Harlequin syndrome). Due to desaturated blood ejected by the heart, the head becomes blue, whereas the lower body remains oxygenated by VA-ECMO. We report on an unusual cardiac manifestation, leading to electrical storm.

**Case summary:**

We present the clinical case of a 55-year-old man. During a minor viral pneumonia, superinfection led to severe ARDS and sepsis-induced refractory cardiogenic shock. Venoarterial extracorporeal membrane oxygenation support was initiated. In progressive respiratory failure, an electrocardiogram (ECG) revealed the onset of ST-segment elevations mirroring hypoxic coronary perfusion. As the mixing zone of blood from the heart and the VA-ECMO was in the ascending aorta, hypoxia was limited to the heart. Ventricular arrhythmias recurred, until ventricular fibrillation remained refractory to defibrillation. A second return cannula was inserted into the jugular vein, and veno-arteriovenous ECMO (V-AV-ECMO) was established. After the venous return was added to the circuit, ventricular fibrillation was defibrillated and sinus rhythm remained stable. Within an hour, ST-elevations receded. Systolic function recovered to normal within 26 days.

**Discussion:**

In severe sepsis-related cardiogenic shock, cardiac output is likely to recover. Venoarterial extracorporeal membrane oxygenation is a potential bridge to recovery. Apart from textbook knowledge, Harlequin syndrome can exclusively cause coronary ischaemia, leading to ST-segment elevations and electrical storm. ECGs reveal ST-elevations for early detection. Isolated cardiac Harlequin syndrome can be overlooked or misinterpreted as result of coronary artery disease, but needs immediate therapy to save the patient’s life (e.g. V-AV-ECMO).

**Learning points**

- Venoarterial extracorporeal membrane oxygenation (VA-ECMO) is a potential bridge-to-recovery in sepsis-induced refractory cardiogenic shock.
- During peripheral VA-ECMO support, severe respiratory failure can lead to differential hypoxia of the body, resulting in a blue, desaturated head as well as upper chest and a pink lower body (Harlequin syndrome).
- Apart from this typical, textbook presentation, differential hypoxia may also specifically affect perfusion of the coronary arteries, causing cardiac ischaemia mirrored by ST-elevations and recurrent ventricular arrhythmias.

## Introduction

In severe sepsis-induced cardiogenic shock refractory to conventional treatment, venoarterial extracorporeal membrane oxygenation (VA-ECMO) can serve as a potential bridge to cardiac recovery. Propensity-weighted analyses of multi-centre studies have shown improved survival rates when compared with conventional treatment.^1^ The current European Society of Cardiology guidelines for acute heart failure recommend considering short-term mechanical circulatory support (Class IIa, Level C).^2^ Unlike infarct-related cardiogenic shock, pneumonia-related sepsis is more likely to be associated with severe acute respiratory distress syndrome (ARDS). During peripheral VA-ECMO support, oxygenated blood is returned via the femoral artery retrograde to the native blood flow. Blood ejected by the ventricle and blood returned from VA-ECMO mix in the aorta (mixing cloud). The position of this mixing zone can vary depending on cardiac output, VA-ECMO blood flow, and systemic vascular resistance^3^. Severe respiratory failure may result in differential hypoxia (Harlequin syndrome). Poorly oxygenated blood deriving from the left ventricle perfuses the ‘upper body’ and fully saturated blood from the VA-ECMO the ‘lower body’. Thus, the head is cyanotic and appears blue, whilst the torso and the legs look pink. As the location of the mixing zone varies, the symptoms of differential hypoxia are heterogeneous. We report an unusual case of ‘cardiac Harlequin syndrome’, specifically affecting coronary perfusion and resulting in ST-segment elevations and electrical storm. This rare phenotype can be easily overlooked, even if blood gases are drawn from the right radial artery.

## Summary figure

**Figure:**
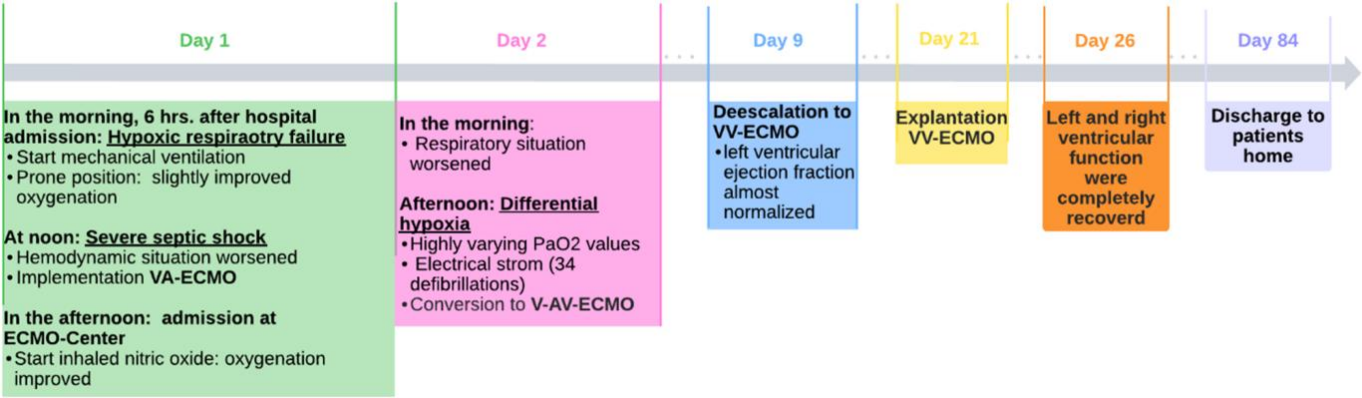


## Case presentation

A 55-year-old man with a history of HLA-B27-positive ankylosing spondylitis was admitted to a rehabilitation hospital due to worsening back pain. He took a JAK inhibitor for polycythaemia vera. Two weeks before, he was in his usual state of health, allowing doing sports and his job. During his stay, he developed a cough and dyspnoea. A SARS-CoV-2 test was positive. A week later, dyspnoea got worse within a day, so he was transferred to a regional hospital at midnight. As pneumonia-induced sepsis was suspected, antibiotics were administered immediately after sampling blood cultures. As acute hypoxaemic respiratory failure aggravated rapidly and non-invasive ventilation failed, he was intubated within hours. Due to persistent hypoxaemia, prone positioning was performed. Severe septic shock [sepsis-related organ failure assessment score (SOFA) 21] was complicated by acute heart failure. Left ventricular function was normal on admission and decreased to an ejection fraction of 11% after 7 h (see Supplementary material online, *Video S1*). Gas exchange remained critical [PaO_2_/FiO_2_ 70 mmHg, positive end-expiratory pressure (PEEP) 18 mbar, FiO_2_ 1.0], and he required escalating doses of vasopressors (1.0 µg/kg/min norepinephrine, 3.3 µg/kg/min dobutamine, 0.00025 international units/kg/min argipressin, and lactate 12 mmol/L), until our mobile ECMO team arrived. Although venovenous ECMO (VV-ECMO) could have been considered for hypoxaemic respiratory failure, VA-ECMO was necessary due to impending circulatory collapse in severe combined cardiogenic and septic shock according to the current transport guidelines.^4^ Our team established peripheral VA-ECMO support via the right femoral artery and the left femoral vein before transferring the patient to our ECMO centre. At arrival, the patient was intubated and on VA-ECMO with high doses of catecholamines. Breath sounded bilaterally similar to rales and rhonchi. The patient had cold extremities, acral cyanosis, and petechiae. Over the next 6 h, vasopressors were reduced (norepinephrine from 0.67 to 0.17 µg/kg/min, argipressin from 0.0003 to 0 IU/kg/min, and dobutamine from 3.3 to 5.0 µg/kg/min). Initial laboratory results showed modestly elevated levels of high-sensitivity troponin T (128 ng/L), rising to 225 ng/L after 12 h with stable, slightly elevated values of creatine phosphokinase-MB (admission 34 ng/mL, after 12 h 33 ng/mL). Inflammatory markers were markedly elevated (procalcitonin 78 ng/mL, interleukin-6 177,019.0 pg/mL). Bronchoalveolar lavage and blood cultures isolated *Streptococcus pyogenes*. A diffuse macular erythroderma was seen as a sign of streptococcal toxic shock syndrome. Within the first day, liver and renal failure developed. Disseminated intravascular coagulation (DIC) leads to petechiae, fast clotting on guide wires (see Supplementary material online, *Video S4*), and bleeding from access sites of ECMO cannula and central lines requiring transfusion of red blood cells. To diagnose DIC, the guideline-endorsed International Society on Thrombosis and Haemostasis (ISTH) overt DIC score was calculated with 5,^5^ reflecting laboratory results of strongly elevated D-dimers (>35 mg/L), activated partial thromboplastin time (53 s) in the absence of heparin, and a low platelet count (47 × 10^9^/L) on Day 1. As plastic surfaces of the VA-ECMO system can further drive consumptive coagulopathy,^6^ we administered 200 IU/h of unfractionated heparin. Heparin-induced thrombocytopenia was ruled out by ELISA testing on Day 5.

With VA-ECMO, optimized mechanical ventilation, and inhaled nitric oxide, oxygenation improved after arrival in our centre (PaO_2_/FiO_2_ 182 mmHg). A computed tomography scan (CT) showed pulmonary consolidation, mainly in the left lower lobe (see Supplementary material online, *Video S5*). CT scans were not characteristic of COVID-19-related ARDS. By the next day, the patient’s respiratory status deteriorated again, and paO_2_ values (arterial gas analysis of blood from the right brachial artery) varied between 55 and 120 mmHg within an hour. Pulse pressure was 12 mmHg. Echocardiography showed a sufficiently opening aortic valve.

**Figure 1:**
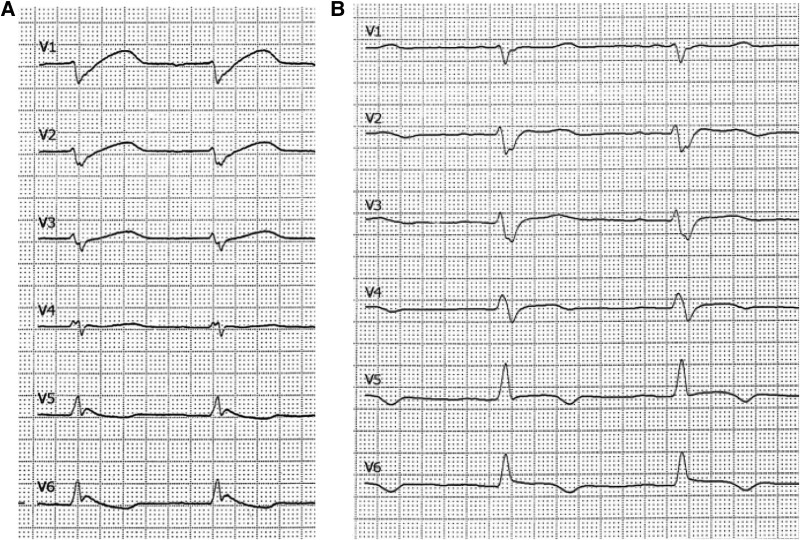
Electrocardiogram with sign for myocardial ischaemia. Hints for myocardial ischaemia. 12-lead electrocardiography revealed ascending ST-elevations as hint for coronary hypoxaemia (*A*), completely decreasing within an hour after the start of V-AV-ECMO (*B*).

Based on these findings, we suspected that the mixing zone was near the brachiocephalic trunk. An electrocardiogram (ECG) showed new ST-segment elevations (*Figure 1*), indicating coronary hypoxaemia and cardiac ischaemia. To tackle differential hypoxia, we decided to insert a second return cannula into the right jugular vein upgrading the circuit to a veno-arteriovenous ECMO (V-AV-ECMO). Thus, saturated blood can enter the venous system and improves oxygenation of the blood ejected by the native heart. Ventricular fibrillation recurred several times and was repeatedly defibrillated. Whilst the cannulation procedure was fastened, ventricular fibrillation became rapidly refractory to defibrillation (cumulative defibrillations: 34). After adding the venous return cannula to the circuit, ventricular fibrillation could be immediately terminated and sinus rhythm was restored. Within an hour, ST-elevations resolved completely (*Figure 1*). Returning arterial-to-venous blood flow ratio was 2:1 and thus sufficient for both sides (paO_2_ 98 mmHg, central venous saturation 75%). During electrical storm and V-AV-ECMO upgrade, chest compressions were necessary, leading to a pneumothorax. Rapidly decreasing tidal volumes and at the same time increasingly frequent chatter of the drainage cannula suggested tension pneumothorax. We immediately inserted a chest tube, which additionally drained the pleural effusion. Its appearance was pus. It showed typical lab results of pleural empyema (pH 6.776, lactate 24 mmol/L, glucose level below detection limit). Cultures of pleural effusion remained negative, probably due to already 30 h of antibiotic therapy. Intrapleural fibrinolysis was performed for 1 week.

Sepsis complicated with DIC was further treated by antibiotic therapy and five cycles of plasmapheresis followed by intravenous immunoglobulins. After 10 days, the patient’s ejection fraction had almost normalized (see Supplementary material online, *Video S2*). By Day 9, the V-AV-ECMO was deescalated to VV-ECMO mode, and by Day 21, the VV-ECMO was removed. Six weeks after his referral to our centre, the patient was weaned from the respirator and transferred to a hospital near to his home. He was treated there for further 5 weeks, before he was finally discharged home. Cerebral performance and systolic left ventricular function recovered fully (cerebral performance category 1, ejection fraction > 55%, Supplementary material online, *Video S3*). Due to high doses of vasopressors the DIC, several toes of both feet were ischaemic even before VA-ECMO cannulation. After further demarcation of the infarcted tissue, amputation of the distal phalanx of the left second toe was performed. The patient required ambulant rehabilitation to regain full mobility and has returned to work.

## Discussion

We report a rare case of ‘cardiac Harlequin syndrome’ during peripheral VA-ECMO support in septic cardiomyopathy. The initial cause of biventricular systolic failure and cardiogenic shock was attributed to septic cardiomyopathy, based on the patient’s history, the isoelectric ST-segments on the initial ECG, rising high-sensitivity troponin T levels, and the rapid recovery within 10 days.^7,8^ COVID-19-related myocarditis was considered but deemed unlikely. Endomyocardial biopsy was not performed due to the risk of life-threatening bleeding from DIC [Bleeding Academic Research Consortium (BARC) score 3a], and cardiac MRI was not possible during ECMO support. Following insertion of the second return cannula, the ST-segment elevations resolved completely. Left ventricular function quickly recovered within 10 days. Given the high likelihood of septic cardiomyopathy, we decided against an acute coronary angiogram in septic shock with coagulation disorder after weighing the risks, benefits, and the low probability of ST-elevation myocardial infarction.

Due to progressive respiratory failure, the blood ejected by the native heart was poorly saturated. The initial dilemma entailed severe cardiogenic shock and hypoxaemia, requiring two different ECMO modes (VA-ECMO vs. VV-ECMO). During peripheral VA-ECMO mode, oxygenation of the blood ejected by the heart can be improved by adjusting mechanical ventilation,^9,10^ e.g. increasing or optimizing PEEP.^11–13^ In severe hypoxaemic respiratory failure with already limited therapeutic options, we initiated inhaled nitric oxygen (iNO). Inhaled nitric oxygen improves arterial oxygenation by decreasing intrapulmonary shunt.^14,15^ Though iNO does not improve mortality in meta-analysis,^16^ it increases the PaO_2_/FiO_2_ ratio and reduces physiologic dead space fraction within 24 h of application.^17,16^ Thus, it is a rescue strategy in ARDS to improve oxygenation. For further improvement in oxygenation, prone positioning could be an option. Prone positioning improves gas transfer and is recommended before initiation of VV-ECMO.^18^ The effect of prone positioning of VV-ECMO patients has not been fully settled yet.^19–21^ Prone positioning is feasible and safe in most VA-ECMO patients. However, we considered the risk of prone positioning in this particular patient as too high due to challenging haemodynamic management and severe DIC with bleeding, whilst the expected beneficial effect did not offset the risk. However, prone positioning could have helped to optimize arterial oxygenation.

As stroke volume decreased, the mixing zone shifted to the ascending aorta around the brachiocephalic artery. For early detection of differential hypoxia, blood has to be drawn from the right arm. It is the blood nearest to the cardiac stroke volume, which can be accessed at a peripheral artery. However, our case demonstrates that measured oxygen tensions may lead astray, when the mixing zone is already around the brachiocephalic trunk and the native stroke volume is very close to the heart. Though coronary perfusion is severely desaturated, blood gases may erroneously indicate sufficient oxygenation. A 12-lead ECG can reveal a cardiac hypoxia by detecting ST-elevations. In consequence of ischaemia, ventricular arrhythmias occur. The resulting high heart rate further aggravates cardiac energy starvation.^22,23^ When differential hypoxia emerges, non-invasive parameters are optimized first: ventilator settings, inotropic agents,^24^ reducing ventricular afterload, or pulmonary fluid overload.^25^ As differential hypoxia remained, we decided to insert a second return cannula in the jugular vein and to convert to V-AV-ECMO. Another option is to drain the blood from the superior vena cava (SVC) instead of inferior vena cava. As central venous oxygen saturation is lower in the SVC, the oxygenator can deliver more oxygen to the body.^26^ Another alternative is central cannulation,^27^ which is a very invasive procedure. In severe sepsis-induced cardiogenic shock, pneumonia-associated ARDS, and DIC, we deemed the effect of SVC drainage on oxygenation as insufficient and we wanted to avoid the invasive central cannulation in the first step.

## Conclusions

The phenotype of differential hypoxia during VA-ECMO support does not necessarily mirror the phenotype described in textbooks. When the mixing zone is between the aortic valve and the brachiocephalic artery, the body appears well perfused, but an isolated hypoxia of the coronary perfusion may occur. It leads to cardiac ischaemia and arrhythmias. In case of a mixing zone around the brachiocephalic artery, cardiac hypoxia is likely to be overlooked. ECG reveals ST-elevation and facilitates early detection. Isolated cardiac hypoxia can be solved by changing to a V-AV-ECMO support.

## Supplementary Material

ytaf059_Supplementary_Data

## Data Availability

The data underlying this article will be shared on reasonable request to the corresponding author.

## Lead author biography

Dr. Maria Heinrich has been a resident in the Department of Cardiology, Pneumology and Internal Intensive Care Medicine at the University Hospital of Regensburg, Germany, since 2020. Her current focus is on acute cardiac care.
